# Synthesis of nano-SiO_2_@PTPEG–VPA copolymer and its effects on early-age cement hydration

**DOI:** 10.1039/d4ra04491f

**Published:** 2024-08-13

**Authors:** Lei Dong, Xin Shu, Qianping Ran

**Affiliations:** a Jiangsu Key Laboratory of Construction Materials, School of Material Science and Engineering, Southeast University Nanjing 211189 China leidong@seu.edu.cn; b State Key Laboratory of High-Performance Civil Engineering Materials, Jiangsu Sobute New Materials Co., Ltd Nanjing 211103 China

## Abstract

Incorporating nano-SiO_2_ particles into cement paste has garnered significant attention for enhancing the performance of hardened cement paste. However, the agglomeration of nanoparticles in the pore solution of cement–water system poses a challenge for cost-effective and efficient applications. Meanwhile, superplasticizers containing phosphate groups exhibit strong complexation with calcium ions and show promise in improving the dispersion performance. This study introduces a surface chemical modification technique to enhance the dispersibility of nano-SiO_2_. Firstly, poly(isoprenyl oxy poly(ethylene glycol) ether-*random*-vinylphosphonic acid) (PTPEG–VPA), a silanized superplasticizer containing phosphate moieties, is copolymerized and chemically grafted onto pristine nano-SiO_2_ surfaces through condensation and silanization processes. The resulting core–shell SiO_2_@PTPEG–VPA nanoparticles are comprehensively characterized using FT-IR spectroscopy, TGA, DLS, TEM, BET surface area analysis, and zeta potential measurements. The results indicate that introducing phosphate moieties improves the dispersion capacity of grafted copolymers, thereby reducing the severe agglomeration of nano-SiO_2_ in solution. Subsequently, the impact of SiO_2_@PTPEG–VPA on cement hydration and early-age strength development is investigated using microcalorimetry and TGA characterization. Finally, a mechanism is proposed to explain the observed retarding effects of grafted PTPEG–VPA on pristine SiO_2_. Overall, this study provides novel insights into the chemical design of nanoparticles, aimed at manipulating cement paste properties.

## Abstract 2

Heat flows of cement hydration with the addition of 0.8 wt% pristine NS and NS@PTPEG–VPA, and corresponding schematic illustration of the seeding effects jointly determined by agglomeration degree and pozzolanic reactivity of NS@PTPEG–VPA.

## Graphical abstract

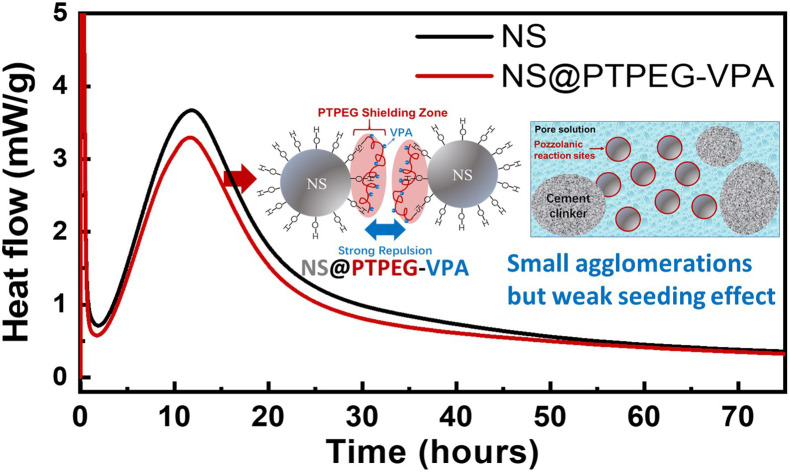

## 1 Introduction

Concrete, as the most widely used construction material, faces the urgent challenge of reducing its environmental footprint while meeting the growing global demand for infrastructure. In particular, the production of cement, which is the binder and primary component of concrete, is receiving considerable attention due to its extremely high CO_2_ emissions. Specifically, global cement production accounts for approximately 7% of global CO_2_ emissions, generating no less than 2.1 billion tons of CO_2_ annually.^1^

To mitigate climate change caused by CO_2_ emissions, innovative strategies have been proposed to reduce cement usage and develop sustainable concrete materials. Among these strategies, partially replacing ordinary Portland cement (OPC) with supplementary cementitious materials (SCMs) has emerged as a promising solution, offering both environmental benefits and improved performance of concrete.^2^ Specifically, SCMs originating from industrial by-products or wastes, such as fly ash, ground granulated blast furnace slag, and silica fume,^3^ provide various benefits for concrete properties, including improved durability, mitigation of alkali–silica reaction, controllable thermal properties, and enhanced workability and finishability. However, these SCMs are generally less reactive than the OPC clinkers, which can lead to delayed hydration and slow strength development of cementitious materials.^4^ To accelerate the hydration process, a series of nanomaterials, such as nano-TiO_2_,^5^ nano-Al_2_O_3_,^6^ nano-SiO_2_ (NS),^7^ nano-Fe_2_O_3_,^8^ nano-NiO,^9^ nano-carbon tubes,^10^ and graphene oxide,^11^ have been utilized, achieving satisfactory early-age strength gain. Among these nanomaterials, NS exhibits immense application potential by improving early strength and other properties. For instance, NS can refine the microstructure of cementitious composites, enhancing the compressive and flexural strengths while also improving the cracking resistance and permeability.^12^ Besides, its pozzolanic activity facilitates the formation of additional calcium silicate hydrate (C–S–H) gel, leading to higher bond strength and improved interfacial transition zones.^13^ The C–S–H gel with increased density also provides better protection against chemical attacks, such as sulfate and chloride ingress, alkali–silica reaction (ASR), and carbonation.^14^ Moreover, its nanoscale dimensions (usually 1–100 nm) facilitate its incorporation into the interfacial transition zone (ITZ), where it acts as a nucleation site for the precipitation of hydration products.^15^ This phenomenon leads to a more refined and homogeneous ITZ, minimizing the occurrence of microcracks and enhancing the overall durability of concrete.

Although NS can improve the overall performance of cementitious materials, especially in terms of the early hydration process and strength development, its practical application is often restricted by high cost, primarily attributed to nanoparticle agglomeration, leading to low efficiency.^16^ Generally, the unique characteristics of nanomaterials, including NS, such as high surface area, enhanced reactivity, and nucleation-promoting properties, can accelerate the cement hydration kinetics. However, the harsh environments in cement paste pore solutions, characterized by high pH and saturated calcium ion concentrations, can cause severe nanoparticle agglomeration, diminishing the effectiveness of nanoparticles.^17^ To improve the dispersion of nano-materials, optimized mixing protocols using high-energy mechanical mixing or ultrasonication^18^ as well as physical dispersants, such as sodium polyacrylate,^19^ poly(acrylic acid), and poly (methacrylic acid),^20^ have been proposed. However, the applied shear force may detach the physically absorbed dispersants from the nano-materials. Besides, the high calcium and salt ion concentration in the cementitious system can cause counterion condensation in the Stern layer of the colloidal surface, changing the structure of the double electric layer and significantly reducing the total polyion charge.^21^ Hence, the alternative chemical dispersion techniques appear more promising as the highly stable, chemically attached dispersing agents can achieve a more uniform dispersion of nano-materials within the cementitious mixture.

Recently, our group developed a novel chemical dispersion technique to synthesize a series of polycarboxylate superplasticizers (PCEs), which were then grafted onto NS particles, forming core–shell nanoparticles.^22,23^ These modified NS particles showed higher stability and dispersion in cementitious environments than unmodified NS particles. Besides, the effects of modified NS on hydration kinetics and pore structure were deeply explored.^24^ However, the dispersity of NS was intrinsically restricted by the grafted PCEs. To improve the dispersing ability of PCEs, phosphated comb polymers with phosphate functionalities were introduced instead of the carboxylate groups commonly found in PCEs.^25,26^ The phosphate groups exhibit strong complexing ability with calcium ions,^27^ effectively improving the adsorption–dispersion performance of superplasticizers while maintaining sulfate and clay tolerance comparable to conventional PCE.^25^ Among the different phosphate groups, compared to the widely used 2-hydroxyethyl methacrylate phosphate (HEMAP),^27^ vinylphosphonic acid (VPA) displays a more compact chemical structure, higher charge density, and better chemical stability and water-solubility.^28^

Based on these advancements, in this study, a phosphate-containing copolymer has been synthesized using VPA monomers and isoprenyl oxy polyethylene glycol (TPEG) macromonomer, which is subsequently grafted onto NS. The composition, microstructure, and dispersity of modified NS are comprehensively characterized. The influence of these NS particles on the early-age hydration of cement paste is thoroughly explored, examining heat release process, microstructure, and macroscopic compressive strength development. Further, the dominating factors influencing the hydration process are elucidated. Overall, the findings of this study provide new insights into the role of phosphate groups in enhancing NS dispersion and affecting hydration process.

## 2 Experiment

### 2.1 Raw materials

Vinylphosphonic acid (VPA), acetic acid, vitamin C, and mercaptopropionic acid with reagent grade were purchased from Sigma-Aldrich. TPEG (*M*_w_ = 2400 g mol^−1^) was provided by Jiangsu Bote, China. Triethoxy vinylsilane (VETO) and hydrogen peroxide (30 wt% solution in water) were purchased from Aladdin and Merck, respectively. Colloidal NS with an averaged 7 nm radius was purchased from Aladdin.

All the cement pastes studied in this paper were prepared by a Benchmark cement P.I 42.5. Table 1 shows its chemical and mineralogical composition measured by XRF and XRD (Rietveld).

**Table 1:** Chemical and mineralogical composition of the cement (wt%)

Chemical composition				Mineralogical composition	
Oxide	XRF/%	Oxide	XRF/%	Phase	XRD (Rietveld)
CaO	60.57	Na_2_O	0.15	C_3_S	40.7
SiO_2_	23.28	K_2_O	0.42	C_2_S	31.5
Al_2_O_3_	4.99	TiO_2_	0.26	C_3_A	2.10
Fe_2_O_3_	3.42	P_2_O_5_	—	C_4_AF	10.5
MgO	2.11	MnO	—	Gypsum	2.3
SO_3_	1.88	LOI	1.83	Quartz	1.10

### 2.2 Synthesis of NS@PTPEG and NS@PTPEG–VPA

The synthesis of NS@PTPEG–VPA or NS@PTPEG is divided into two procedures, including the copolymerization of superplasticizer copolymers and the following grafting of copolymers onto the surface of NS. Typically, in a 500 mL Schlenk flask with a magnetic bar, 204 g isoprenyl oxy polyethylene glycol (–[CH_2_–CH(CH_2_–CH_2_–O–(–CH_2_–CH_2_–O)_*n*_–H)–]_*b*_–), and 0.5 g of acetic acid were mixed, and 2.02 g of hydrogen peroxide and 4.54 g of triethoxy vinylsilane were added, yielding solution 1. Solution 2 containing 0.81 g of vitamin C, 0.84 g of mercaptopropionic acid, and 51 g of water was added dropwise into solution 1. Subsequently, NaOH was added to keep the alkaline condition. For PTPEG–VPA, an additional 18.6 g of VPA was added into solution 2.

Subsequently, 7.8 g of the synthesized copolymer was added dropwise into 100 g of NS, followed by a water bath at 40 °C with mechanical stirring. Then, the obtained water solution was dialyzed in deionized water for around 3 days until no obvious signals of organic residuals in water could be detected by the total organic carbon (TOC) instrument.

### 2.3 Characterization of NS@PTPEG and NS@PTPEG–VPA

Fourier transform infrared (FT-IR) spectroscopy (Nicolet 5700, Thermo Fisher) was used to characterize the copolymers grafted on the surface of NS. The core–shell ratio of NS was determined by thermogravimetric analysis (TGA; MDSC2910, American TG Company). The mean size and size distribution of NS were determined by dynamic light scattering (DLS; CGS-3, ALV CO.) using a wide-angle light scattering instrument, which can monitor particles in the size range 1 nm to 5 μm. The NS solution was treated in an ultrasonic bath for 2 min and then diluted to 1 mg g^−1^ using deionized water for DLS measurements. The surface charge and zeta potentials of NS in water of various pH were measured with a DT310 zeta potential analyzer. The microstructure of NS was characterized by transmission electron microscopy (TEM; Talos F200X, Thermo Fisher Scientific). Brunauer–Emmett–Teller (BET) analysis (Micromeritics APSP 2460) with 2 MP gas adsorption analyzer at 77 K was adopted to investigate the specific area of pristine and modified NS in a solid state prepared from an aqueous solution. The organic residuals in water were measured by a TOC analyzer (Analytik Jena AG multiN/C 3100).

### 2.4 Preparation of cement pastes

A water–cement ratio (w/c) of 0.4 was adopted for the desirable workability of NS/cement pastes, and the dosage of pristine and modified NS was set to 0.4 or 0.8 wt% of cement. The mixed proportions of all the samples are listed in Table 2. All the samples were magnetically mixed for 3 min and poured into a cubic mold with a side length of 10 mm. After being placed in an environment with a temperature of 20 °C with relative humidity above 90% for 1 day, the specimens were demolded and cured for another 2 days to obtain the hardened cement paste. The cubic hardened cement paste (10 mm × 10 mm × 10 mm) samples were further used for compressive strength tests. The samples were also crushed and grounded into powers for TGA.

**Table 2:** Mix proportions of various NS-doped cement paste samples (mass in a unit of g)

Dosage (wt%)	Sample	Cement (g)	Water (g)	NS (g)
0.4	NS/cement	100	40	0.4
	NS@PTPEG/cement	100	40	0.4
	NS@PTPEG–VPA/cement	100	40	0.4
0.8	NS/cement	100	40	0.8
	NS@PTPEG/cement	100	40	0.8
	NS@PTPEG–VPA/cement	100	40	0.8

### 2.5 Characterization of cement hydration

TAM Air thermal activity micro-calorimeter was applied to measure the hydration heat of the cement pastes. The cement powder premixed with 0.4 or 0.8 wt% NS was added into water (w/c = 0.4) and stirred for 2 min to form a paste, and then about 20 g of paste was weighed. The hydration heat test was started 10 min after the powder came in contact with water and recorded every 10 s. During the test, both the experimental temperature and the ambient temperature were maintained at 20 °C.

The mineral compositions of cement paste were characterized by TGA (MDSC2910, American TG Company). For TGA, the calcium hydroxide (CH) content of the sample was estimated from eqn (1).^29^1
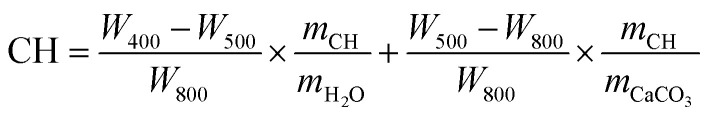
where *W* denotes the recorded weight at specific temperatures; 400 and 500 represent the weight loss temperatures of CH at 400 °C and 500 °C, respectively, while 500 and 800 are the weight loss temperatures of CaCO_3_ at 500 °C and 800 °C, respectively; *m*_CH_, *m*_H_2_O_, and *m*_CaCO_3__ are the molecular mass of CH, H_2_O, and CaCO_3_, respectively.

### 2.6 Compressive strength test

Compressive strength tests were carried out to measure the compressive strength of control sample and NS/cement paste at the age of 24 h and 72 h, according to GB/T 50081-2019. The compressive strength of each cement sample was measured three times to obtain the average value.

## 3 Results and discussion

### 3.1 Synthesis and characterization of NS@PTPEG and NS@PTPEG–VPA

**Fig. 1:**
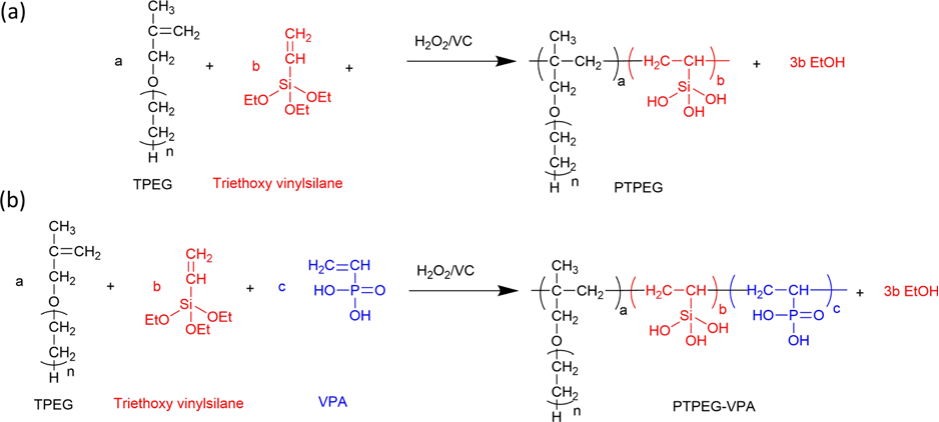
Synthetic route and chemical structure of (a) PTPEG and (b) PTPEG–VPA.

Fig. 1 shows the synthetic route of NS@PTPEG and NS@PTPEG–VPA, which includes two steps. The first step involves the free radical copolymerization of TPEG, VETO, and VPA monomers. TPEG with bulky side chains is widely employed in superplasticizers due to the steric hindrance caused by its adsorption on cement particle surfaces, thereby enhancing the dispersity of cement. VETO is incorporated into the copolymers owing to its strong anchoring effect. Specifically, the introduced ethoxysilane in the polymer structures undergoes hydrolysis under alkaline conditions, resulting in Si–OH that can subsequently condensate with the silanol groups on the colloidal silica surface. This process facilitates the chemical modification of silica surfaces *via* the synthesized copolymers.

VPA, a commercial monomer containing a phosphonic acid group, exhibits a higher charge density under alkaline conditions compared to the carboxylic groups typically used in cement superplasticizers. The strong electrostatic effect and Ca^2+^ complexity of phosphate groups significantly contribute to excellent dispersion performance.

**Fig. 2:**
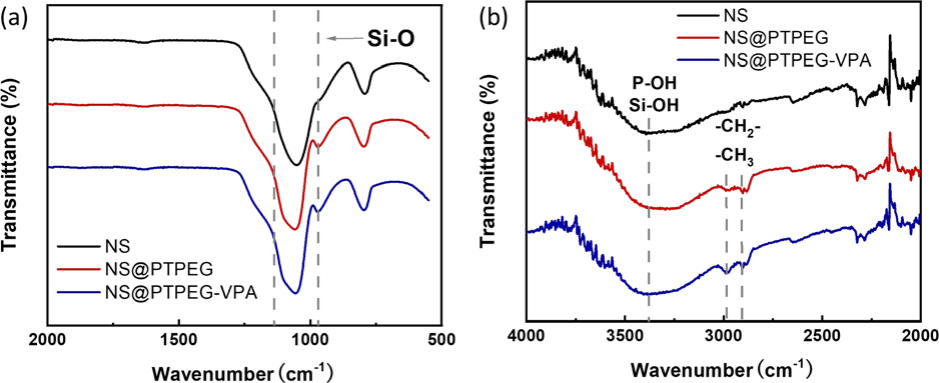
(a) FTIR spectra of pristine NS, modified NS@PTPEG, and NS@PTPEG–VPA. (b) Magnified bands in the range of 4000–2000 cm^−1^.

Fig. 2 presents the FTIR spectra of pristine NS and modified NS@PTPEG and NS@PTPEG–VPA. The broad peaks around 3400 cm^−1^ in all the samples correspond to the stretching vibration of O–H, and the pronounced peak in NS@PTPEG–VPA spectrum may be ascribed to the introduced VPA moieties containing phosphonic acid groups. Both NS@PTPEG and NS@PTPEG–VPA exhibit strong bands at 2984 and 2904 cm^−1^, which are attributed to the asymmetric –CH_2_–, symmetric –CH_3_, and –CH_2_– stretching vibrations in grafted copolymers.^30^ Besides, the strong band in the range of 1110–970 cm^−1^ corresponds to the stretching vibration of Si–O. Compared to the pristine NS samples, the intensity of this band is increased by surface modification, which may be attributed to the silanol groups in VETO moieties of the polymers. As the dialysis process can remove all physically absorbed unreacted monomers and polymers, the observed characteristic peaks confirm that the synthesized copolymers are chemically bound to NS particles.

**Fig. 3:**
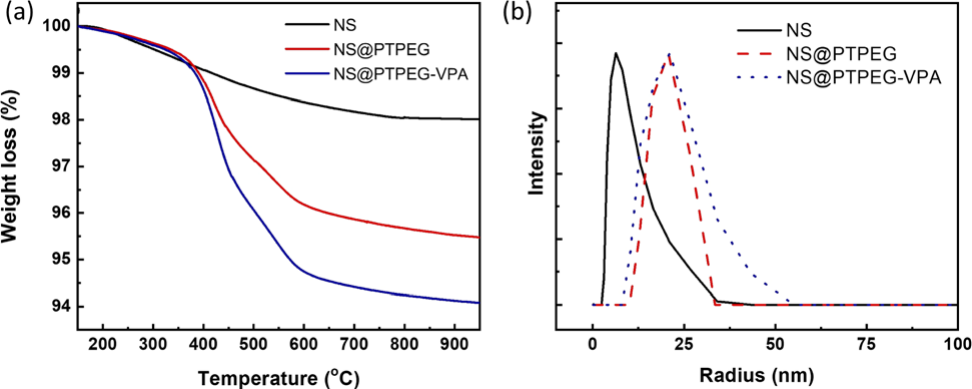
(a) TGA curves and (b) DLS particle size distribution curves of NS, NS@PTPEG, and NS@PTPEG–VPA.

Fig. 3(a) compares the TGA results of pristine NS, modified NS@PTPEG, and NS@PTPEG–VPA, aimed to evaluate the composition of inorganic–organic composite particles. The first decomposition stage is detected from 100 °C, which is likely due to the evaporation of free water molecules from the surface of NS. A more substantial weight loss occurs in the temperature range of 300–600 °C, corresponding to the thermal oxidation and pyrolysis of polymeric shells. Above 600 °C, the TGA curves become nearly flat, indicating the completion of organic shell decomposition.

In this study, the residual mass of modified NS@PTPEG or NS@PTPEG–VPA at 750 °C is regarded as the core mass of NS, while the weight loss during 300–600 °C is used to estimate the content of grafted polymeric shells. Therefore, the mass ratios of shell to core for NS@PTPEG and NS@PTPEG–VPA are calculated as 4.1 and 5.6 wt%, respectively. Considering that the feeding mass proportion of copolymers to NS was 7.8% in the synthesis process, the grafting efficiencies of the two copolymers are estimated to be 53% and 72%, respectively. The higher grafting efficiency of NS@PTPEG–VPA is likely due to the enhanced adsorption of copolymers containing phosphate groups onto the NS surface.

DLS technique was utilized to investigate the influence of grafted copolymers on the dispersed state of NS, and the results are presented in Fig. 3(b). The DLS analysis of pristine NS reveals a mean hydrodynamic radius of 7.5 ± 3.7 nm, with an extremely narrow particle size distribution, which is consistent with the manufacturer's specified value of approximately 7 nm, primarily in a mono-disperse state. Upon chemical modification, the mean hydrodynamic radius of NS@PTPEG and NS@PTPEG–VPA are significantly increased to 20.7 ± 7.8 and 24.0 ± 14.4 nm, respectively. This increase in hydrodynamic radius is attributed to the grafted copolymers on the NS surface. Moreover, the different types of grafted copolymers and their varying grafting efficiencies significantly affect the stretching state of polymeric shells in aqueous solution, leading to differences in mean particle size and particle size distribution. In conclusion, the DLS results confirm the successful chemical grafting process.

**Fig. 4:**
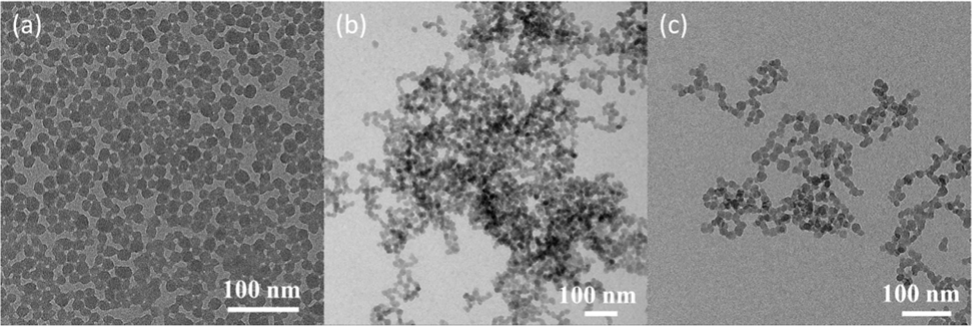
TEM micrographs of (a) NS, (b) NS@PTPEG, and (c) NS@PTPEG–VPA.

To further explore the effects of polymeric shells on NS dispersion, the TEM micrographs of pristine and modified NS in aqueous solution are shown in Fig. 4. The images reveal closely packed clusters of unmodified NS particles, whereas the particles of modified NS@PTPEG and NS@PTPEG–VPA are well dispersed in the aqueous solution. Although some agglomerations of modified NS particles are observed within the images, the majority of modified NS particles are more uniformly distributed. This observation is in excellent agreement with the DLS characterization results, indicating the improved dispersion stability due to the presence of grafted polymers.

**Fig. 5:**
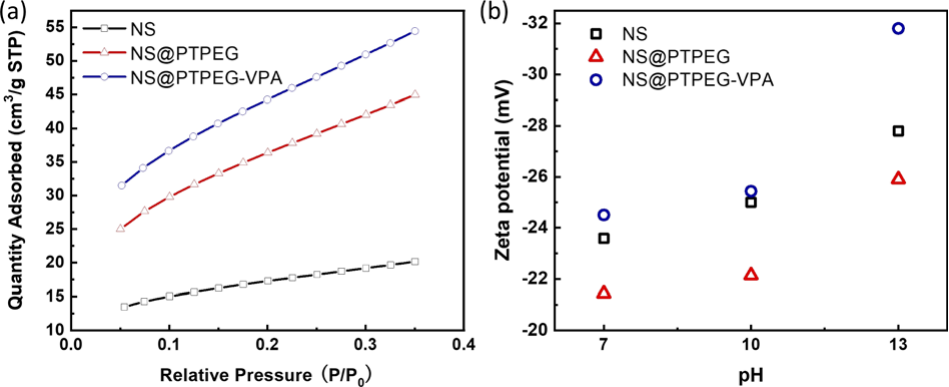
(a) BET isotherms and (b) zeta potentials of NS, NS@PTPEG, and NS@PTPEG–VPA at different pH values.

Fig. 5(a) displays the BET isotherms of solid pristine and modified NS precipitated in simulated pore solution. According to the BET results, the measured specific surface areas of pristine NS, NS@PTPEG, and NS@PTPEG–VPA are 61.50, 132.32, and 161.19 m^2^ g^−1^, respectively. The specific surface area of modified NS is significantly larger than that of pristine NS, indicating the improved dispersity of NS after chemical modification. This change in BET value is also in good agreement with the TEM results.

Remarkably, the specific surface area of NS@PTPEG–VPA is 1.2 times higher than that of NS@PTPEG, indicating additional dispersion effects from the introduced phosphate groups in the polymer structure. According to the mass ratio of monomers used in the synthesis, approximately 8 wt% VPA monomers were incorporated into the copolymers. Assuming equal monomer conversion rates, the 8 wt% poly(vinyl phosphonic acid) randomly distributed within the copolymers can ionize in the highly alkaline cement paste environment. Compared to the interactions involving the deprotonation of silanol groups on the silica surface, the resultant strong electrostatic interaction among the dispersed NS@PTPEG–VPA, despite the ionic bridging triggered by calcium ions in concrete pore solution,^31^ likely contributes to the improved dispersion stability and reduced NS agglomeration. However, the significant increase in specific surface area is mainly attributed to the effective steric hindrance caused by the long side chain of grafted copolymers.

To further analyze the surface charge states of pristine and modified NS, the zeta potentials were measured. These values are presented in Fig. 5(b). All zeta potentials are negative in alkaline solutions, indicating the presence of negative ion groups on the NS surface. In an aqueous solution with pH = 13, NS@PTPEG–VPA exhibits the most negative zeta value, which is consistent with the previous BET isotherm analysis. With p*K*_a1_ = 2.16, p*K*_a2_ = 7.21, and p*K*_a3_ = 12.3 for phosphates groups,^32^ nearly all protons in the phosphates groups are dissociated in the aqueous phase, leading to a more negatively charged particle surface. Interestingly, the sequence of zeta potentials changes under lower pH conditions: NS@PTPEG–VPA ∼ NS < NS@PTPEG. This can be ascribed to the competition between the contributions from ionic phosphate groups and the shielding effect of PTPEG. In aqueous solutions with pH below 10, the polymeric shells may act as a shielding layer, inhibiting the dissociation of abundant silanol groups on the NS surface. Consequently, NS@PTPEG with nonionic polymeric shells exhibits the least negative zeta potential value.

An illustration of nanoparticle dispersion stability in solution can be established based on the widely used Derjaguin–Landau–Verwey–Overbeek (DLVO) theory.^33^ According to this theory, the potential between approaching particle systems is governed by both the attractive van der Waals force and electrostatic repulsive force. For pristine NS, the inter-particle potential consists of attractive van der Waals potential and electrostatic repulsive potential. In concrete pore solution, the positively charged calcium ions are adsorbed onto negatively charged silanol groups on the NS surface, reducing the electrostatic repulsive force among the NS particles. Consequently, the potential is dominated by the van der Waals attractive force, causing a severe nanoparticle agglomeration.

Meanwhile, due to the introduced steric hindrance of grafted polymeric shells, an additional steric hindrance potential term is added to the overall potential expression^34^ when the distance between nanoparticles reaches the overlapped shielding zone. This extra factor can compensate for the van der Waals attractive force, thereby reducing the degree of agglomeration. To optimize the total inter-particle potential for minimizing the agglomeration, thicker polymeric shells with stronger electrostatic characteristics are preferred. Therefore, NS@PTPEG–VPA is speculated to disperse more uniformly in the cement paste than NS and NS@PTPEG.

### 3.2 Cement hydration characterization

**Fig. 6:**
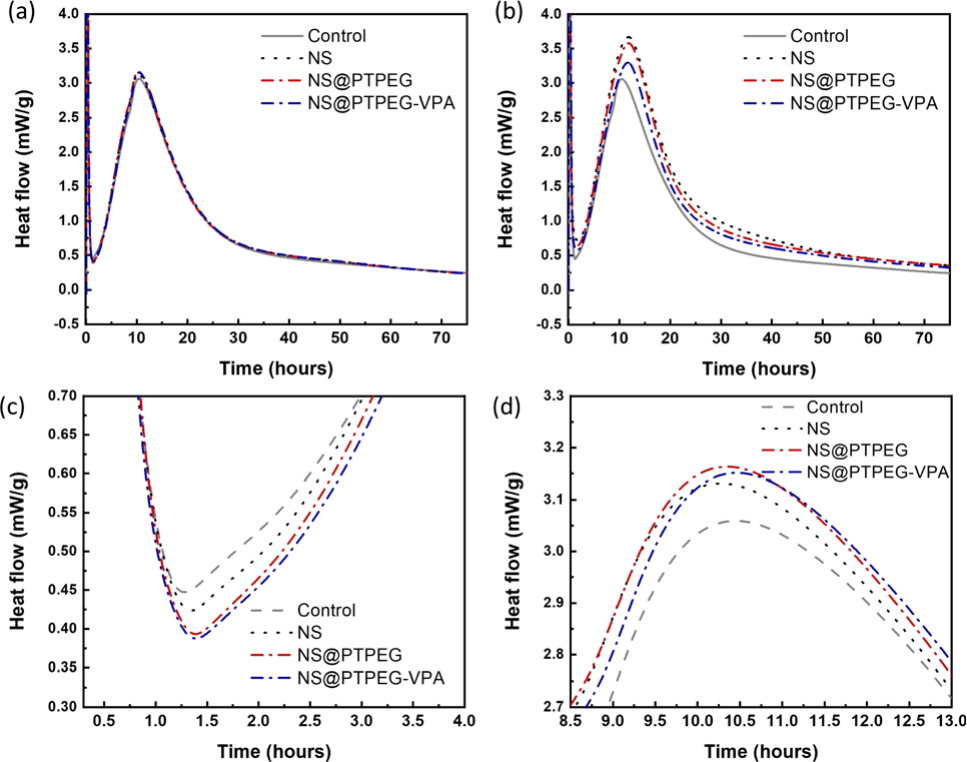
Heat flow of cement hydration with the addition of (a) 0.4 wt% and (b) 0.8 wt% pristine and modified NS in the entire duration of 72 h. Magnified heat flow curves (c) at the initial points of the acceleration stage and (d) around the main hydration peak with a 0.4 wt% dosage.

The exothermic heat flow curves of cement pastes with a fixed NS dosage of 0.4% and 0.8% by mass of cement are shown in Fig. 6. According to the heat flow curves, the maximum heat flow, cumulative heat, and occurrence time of the main hydration peak are summarized in Table 3.

**Table 3:** Maximum heat flow, cumulative heat, and occurrence time of main hydration peak for cement pastes with added pristine and modified NS

Dosage (wt%)	Sample	Maximum heat flow (mW g^−1^)	24 h cumulative heat (J g^−1^)	72 h cumulative heat (J g^−1^)	Time (h)
—	Control	3.06	162.69	239.28	10.45
0.4%	NS/cement	3.13	198.90	277.47	10.27
	NS@PTPEG/cement	3.16	172.67	251.63	10.40
	NS@PTPEG–VPA/cement	3.15	169.46	249.13	10.46
0.8%	NS/cement	3.67	230.15	343.69	11.83
	NS@PTPEG/cement	3.58	220.03	326.12	11.83
	NS@PTPEG–VPA/cement	3.30	198.97	296.42	11.67

Generally, the cement hydration process progresses through several stages: initial dissolution stage, induction period, acceleration stage, and deceleration stage.^35^ The initial dissolution stage occurs within a few minutes after mixing, followed by the induction period where a protective layer is formed on the surface of reacting cement minerals. After this period with minimum heat flow rate, the hydration is rapidly accelerated. During the acceleration stage, C_3_S in cement clinker is converted into massive C–S–H gels and portlandite. As hydration is gradually transformed from a reaction-controlled to a diffusion-controlled process, the heat flow becomes maximum and then decreases, indicating entry into the deceleration stage.

Compared with the control sample, NS has a significant enhancement effect on the cement hydration rate, even at a relatively low dosage of 0.4 wt%. The maximum heat flow increases from 3.06 mW g^−1^ to 3.13 mW g^−1^, and the time of occurrence of the main hydration peak is shortened. Moreover, owing to this acceleration effect, the cumulative heat of cement hydration within 24 h and 72 h is increased by 22% and 16%, respectively. Notably, as shown in Fig. 6(c), the addition of NS slightly delays the start of the acceleration period, and a higher slope of curves in the acceleration period can be observed.

When the dosage is increased to 0.8 wt%, the maximum heat flow of all investigated NS samples is dramatically increased, accompanied by an enhancement in cumulative heat at both 24 h and 72 h. NS@PTPEG–VPA/cement exhibits the smallest increment, which is ascribed to the significant retarding effects of the introduced phosphate groups.^36,37^ Besides, the occurrence time of the main hydration peak is significantly delayed by 1.3–1.4 h, indicating an enhanced but delayed exothermic process.

Based on the changes in the maximum heat flow, peak occurrence time, and exothermic curve slope, the observed acceleration effect can be explained as follows. The nanoparticles, such as NS used in this study, serve as nucleation seeds for the precipitation of hydrates. According to previous studies,^38^ this seeding effect of NS is caused by the nucleation of C–S–H seeds with additional surfaces. In other words, the pozzolanic reaction between NS and calcium hydroxide generates more nucleation sites of C–S–H seeds. These tiny C–S–H seeds can then diffuse and grow in confined spaces, such as inside the capillary pores of paste, accelerating the hydration process. By contrast, without NS, the precipitation of C–S–H gels is limited to the surface of cement clinker particles. Besides, the two commonly reported hydration peaks: silicate peak and sulfate depletion peak, cannot be easily distinguished in this study. These characteristics are determined by the intrinsic properties of cement, which are outside the scope of this study.

The influence of NS polymeric shells on cement hydration is also evident at a dosage of 0.4 wt%. Compared with the control sample without any NS addition, all NS-added cement pastes exhibit a similar increase in the maximum heat flow. Besides, the occurrence time of the main peak is slightly delayed by 0.13–0.20 h. Considering the recorded starting time of the acceleration stage, the estimated slope of the exothermic curve in this stage follows the sequence: NS/cement ∼ NS@PTPEG/cement ∼ NS@PTPEG–VPA/cement > control.

Based on the above discussion, the seeding effect of NS is influenced by two factors: its degree of agglomeration and pozzolanic reactivity. From the perspective of agglomeration, the grafted copolymers improve the dispersity of NS, thereby reducing the degree of agglomeration. Hence, NS with a larger specific surface area produces more C–S–H seeds in the pores, accelerating the precipitation of C–S–H hydrates. On the other hand, from the perspective of pozzolanic reactivity, only the silanol groups on the nanoparticle surface can react with calcium hydroxide. To some extent, the grafted copolymers may act as a shielding layer, limiting the contact between calcium hydroxide in the solution and NS. Therefore, the reactive surfaces of NS are reduced, causing a decrease in the pozzolanic reaction rate. These two factors can synergically affect the seeding process, resulting in similar curve slopes of pristine and modified NS. Besides, for the samples with modified NS, the cumulative heats within 24 h and 72 h are approximately 170 J g^−1^ and 250 J g^−1^, which are higher than that of the control sample but lower than that of NS/cement. Therefore, the enhancement effect of the investigated nanoparticles for cement hydration heat follows this order: NS > NS@PTPEG ∼ NS@PTPEG–VPA. Since the order of degree of agglomeration for the investigated nanoparticles is NS > NS@PTPEG > NS@PTPEG–VPA, the grafted copolymers primarily reduce the pozzolanic reactivity and suppress the released hydration heat during the acceleration stage, offsetting the acceleration effects from improved dispersity.

**Fig. 7:**
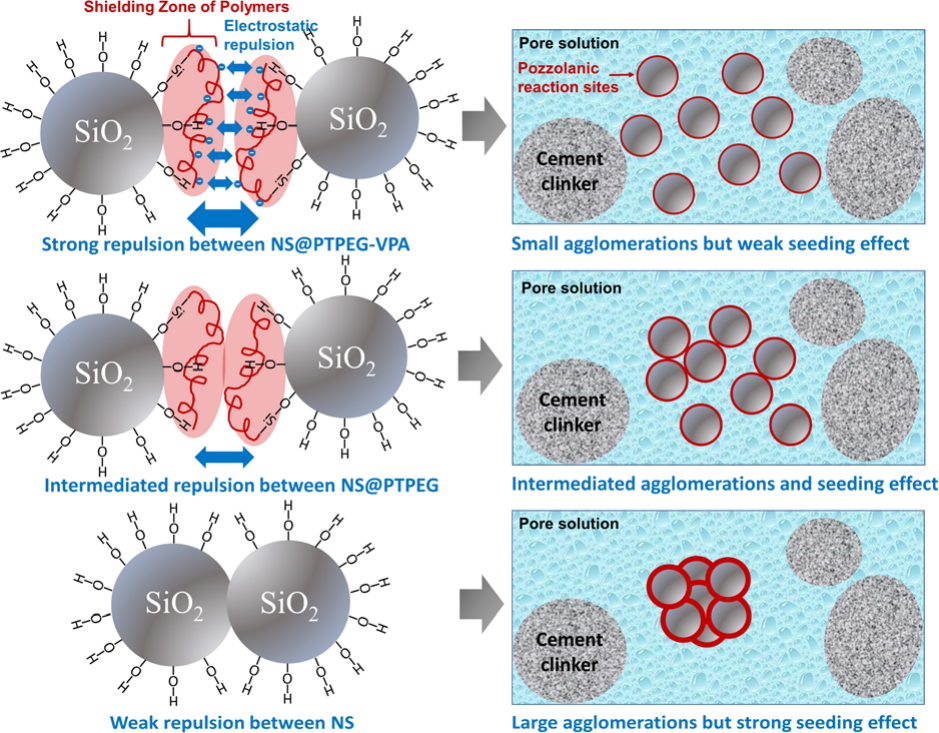
Schematic illustration of the different seeding effects jointly determined by agglomeration degree and pozzolanic reactivity of NS.

The reduced pozzolanic reactivity is more pronounced for NS@PTPEG–VPA as the dissociated phosphate groups exhibit strong complexing ability with calcium ions, leading to the formation of precipitated calcium phosphate, which can retard the cement hydration and pozzolanic reaction.^39^ Moreover, a similar trend is observed at a higher dosage of 0.8 wt%. Based on the above discussion, the observed seeding effect of modified NS is more affected by its pozzolanic reactivity rather than its agglomeration degree. The mechanism is depicted in Fig. 7. Notably, when the shell-to-core ratio of NS exceeds 10 wt%, the cement hydration heat is dramatically increased, indicating that the effect of decreased agglomeration degree begins to dominate the hydration process.^24^

**Fig. 8:**
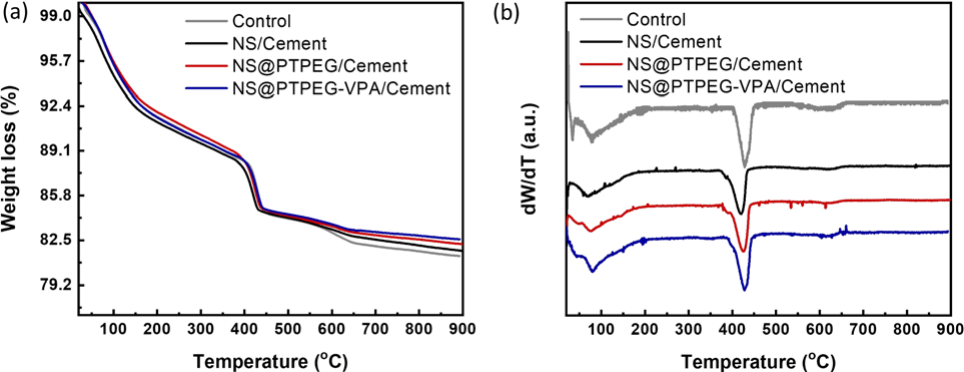
(a) TGA curves and (b) differential TGA curves under the addition of 0.8 wt% pristine and modified NS at 72 h.

To further quantify the content of calcium hydroxide in the hydration products, TGA was conducted on the cement paste at 72 h, and the results are shown in Fig. 8(a). Further, the content of calcium hydroxide can be determined through the differential mass loss between 400 and 500 °C, as depicted in Fig. 8(b). Generally, the TGA curves exhibit several distinct weight loss stages: the first stage between 50 and 200 °C corresponds to the loss of evaporable water as well as the dehydration of C–S–H gel and ettringite; the second stage between 400 and 500 °C is caused by the dehydration of calcium hydroxide; the last stage between 500 and 800 °C is ascribed to the decarbonization of calcium carbonate. Therefore, the calcium hydroxide contents in the control, NS/cement, NS@PTPEG/cement, and NS@PTPEG–VPA/cement are estimated to be 23.6 wt%, 19.4 wt%, 21.6 wt%, and 20.9 wt%, respectively.

The amount of calcium hydroxide, a hydration product derived from C_3_S and C_2_S, is typically governed solely by the hydration degree. However, the addition of NS causes another chemical process: the pozzolanic reaction between NS and calcium hydroxide. Consequently, calcium hydroxide is continuously consumed as the pozzolanic reaction progresses. Given the estimated calcium hydroxide contents of different pastes, the pozzolanic reaction is significant at 72 h, which corresponds to a relatively late stage of the hydration process. Compared with pristine NS, the modified NS exhibits a slower pozzolanic reaction process, which is consistent with the hydration heat results.

### 3.3 Compressive strength analysis

**Fig. 9:**
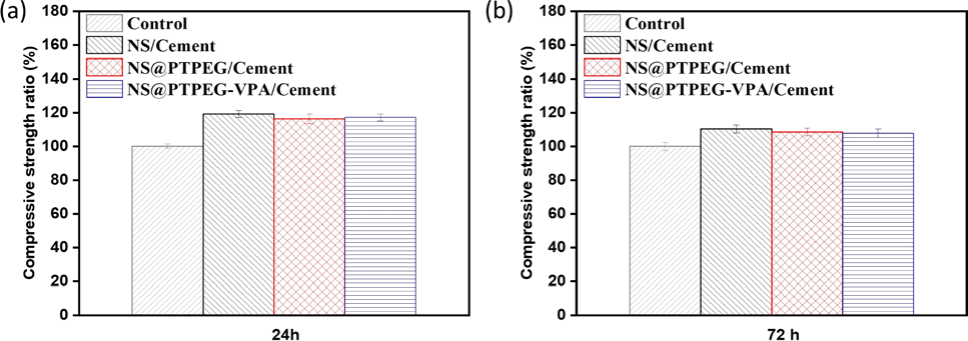
Relative compressive strengths of cement pastes with 0.8 wt% addition of pristine NS, modified NS@PTPEG, and NS@PTPEG–VPA at (a) 24 h and (b) 72 h.

Fig. 9 shows the relative compressive strength development of cement pastes under the addition of 0.8 wt% pristine and modified NS. Generally, compared to the control sample, all pastes containing NS exhibit higher compressive strength at different curing times. This increase is the most pronounced in the sample with pristine NS. Specifically, the compressive strength is increased by approximately 19% and 10% at 24 h and 72 h, respectively. The difference in strength between the control and NS-added sample gradually diminishes as the hydration process progresses. This observation is in excellent agreement with the recorded cumulative hydration heat, as the existing literature suggests a linear relationship between the compressive strength and cumulative heat release.^40^

Notably, the samples with modified NS show less pronounced early strength enhancement compared to pristine NS, which is supported by the lower cumulative hydration heat detected in the early stages. However, during later stages, the compressive strength approaches that of pristine NS-containing sample, which is attributed to a delayed pozzolanic reaction between modified NS and calcium hydroxide. In addition to the modified hydration degree, the incorporation of NS may increase the average length of silicate chains in the produced C–S–H gels,^41^ further contributing to enhanced compressive strength.

**Fig. 10:**
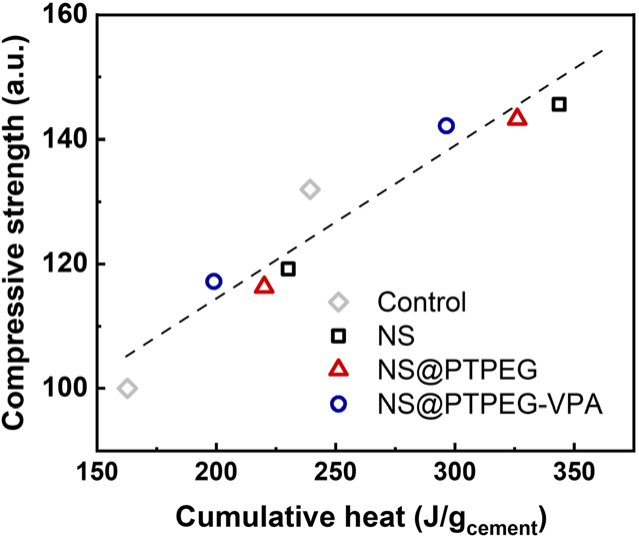
Measured compressive strength *versus* cumulative heat release, normalized by the mass of cement.

To investigate this correlation in the hydration system with NS, the relationship between compressive strength and cumulative heat release is explored, as depicted in Fig. 10. A strong linear correlation is observed, with the majority of data points falling within the acceptable range of the linear fit line (*R*^2^ = 0.92). These findings imply that the mechanical properties of cement pastes containing pristine and modified NS can be predicted from the collected heat evolution data. This correlation provides a valuable supplement to the strength development tests.

## 4 Conclusions

In summary, two silanized polymers: PTPEG and PTPEG–VPA, containing pendant phosphate groups, were synthesized by solution radical polymerization using TPEG and VPA as monomers. These copolymers were subsequently chemically grafted onto the surface of colloidal NS. FTIR spectroscopy, TGA, zeta potential analysis, DLS, TEM, and BET characterizations indicated a grating efficiency of approximately 50–70%, and modified NS particles with shell-to-core mass ratios of around 4–5% were obtained. Owing to the strong steric hindrance from TPEG moieties and electrostatic repulsion from VPA moieties, the NS modified with PTPEG–VPA exhibited improved dispersity and reduced nanoparticle agglomeration in an aqueous solution.

Further, the effects of the prepared NS on cement hydration at early stages were investigated, and the modified sample displayed a weaker acceleration effect than the pristine one. Generally, the early hydration process is preferentially governed by the reduced pozzolanic reactivity caused by grafted copolymers, rather than merely impacting the agglomeration degree. The TGA results also verified the distinguishable pozzolanic reactivities of NS and hydration degrees of the cement paste. A positive correlation between the released hydration heat and compressive strength of cement pastes was observed. Furthermore, the disparity in strengths between cement pastes with pristine and modified NS decreased over time due to the pozzolanic reaction delayed by grafted copolymers. These results underscore the role of copolymers containing phosphate groups in regulating cement hydration and early-stage strength evolution.

## Data availability

The authors confirm that the data supporting the findings of this study are available within the article.

## Author contributions

Lei Dong: conceptualization, methodology, investigation, formal analysis, data curation, writing – original draft, writing – review & editing, funding acquisition. Xin Shu: conceptualization, methodology, resources. Qianping Ran: supervision, resources.

## Conflicts of interest

There are no conflicts to declare.
